# Seco-Taondiol, an Unusual Meroterpenoid from the Chilean Seaweed *Stypopodium flabelliforme* and Its Gastroprotective Effect in Mouse Model

**DOI:** 10.3390/md13041726

**Published:** 2015-03-30

**Authors:** Carlos Areche, Julio Benites, Alberto Cornejo, Lina M. Ruiz, Olimpo García-Beltrán, Mario J. Simirgiotis, Beatriz Sepúlveda

**Affiliations:** 1 Departamento de Química, Facultad de Ciencias, Universidad de Chile, Santiago 8320000, Chile; 2 Facultad de Ciencias de la Salud, Universidad Arturo Prat, Casilla 121, Iquique 1100000, Chile; E-Mail: juliob@unap.cl; 3 Escuela de Tecnología Médica, Facultad de Medicina, Universidad Andrés Bello, Santiago 8370186, Chile; E-Mail: alberto.cornejo@unab.cl; 4 Facultad de Ciencias de la Salud, Universidad Autónoma de Chile, Santiago 8910132, Chile; E-Mail: limarui_hinca@yahoo.com; 5 Facultad de Ciencias Naturales y Matemáticas, Universidad de Ibagué, Carrera 22 Calle 67, Ibagué 730001, Colombia; E-Mail: jose.garcia@unibague.edu.co; 6 Laboratorio de Productos Naturales, Departamento de Química, Facultad de Ciencias Básicas, Universidad de Antofagasta, Antofagasta 1240000, Chile; E-Mail: mario.simirgiotis@uantof.cl; 7 Departmento de Ciencias Químicas, Facultad de Ciencias Exactas, Universidad Andrés Bello, Quillota 980, Viña del Mar 2520000, Chile; E-Mail: bsepulveda@uc.cl

**Keywords:** *Stypopodium flabelliforme*, meroditerpenoids, gastroprotective, gastric ulcer, seaweed

## Abstract

Ten known meroterpenoids and the new meroterpenoid **7** were isolated from the Chilean seaweed *Stypopodium flabelliforme* as their acetylated derivatives. Furthermore, the known metabolite taondiol has been isolated for the first time from this species. The molecular structure of the new metabolite was determined by spectroscopic methods based on 1D- and 2D-NMR. Isolation of **7** represents a key step toward a better understanding of the biogenesis of this class of meroterpenoids. Among the meroditerpenoids isolated, stypodiol, isoepitaondiol, epitaondiol and sargaol exhibited gastroprotective activity on the HCl/Ethanol-induced gastric lesions model in mice. Regarding the mode of gastroprotective action, the activity of epitaondiol was reversed significantly when animals were pretreated with indomethacin, *N*-ethylmaleimide and *N*-nitro-l-arginine methyl ester (L-NAME) suggesting that prostaglandins, sulfhydryl groups and nitric oxide are involved in their mode of gastroprotective action. In the case of sargaol the gastroprotective activity was attenuated with indomethacin and *N*-ethylmaleimide, which suggests that prostaglandins and sulfhydryl groups are also involved in the mode of action using this model.

## 1. Introduction

*Stypopodium flabelliforme* belongs to the family Dictyotaceae (Phaeophyta) and has shown to be a rich source of several polycyclic meroditerpenoids, chromenes and plastoquinones such as epitaondiol, isoepitaondiol, 2β,3α-epitaondiol, epystypodiol, stypodiol, stypotriol, 4-chlorostypotriol, 14-ketostypodiol, sargaol, flabellinol, flabellinone, stypotriolaldehyde, stypohydroperoxide, stypoldione, geranylgeranylbenzoquinones, fucoxanthin and iditol [1,2,3,4,5,6]. Species such as *Stypopodium flabelliforme* are found in Easter Island (Chile) and Long Island (Papua new Guinea) [1,4]. These compounds previously isolated have displayed interesting biological activities such as ichthyotoxic, microtubule assembly inhibitor, anti-inflammatory, sodium channel blockers, radical-scavenging, insecticidal, antimicrobial, negative ionotropic, gastroprotective, antiviral activities, besides anti-proliferative activity to Caco-2 (human colorectal adenocarcinoma), RBL-2H3 (rat basophilic leukemia), V79 (Chinese hamster fibroblasts), SH-SY5Y (human neuroblastoma) and RAW.267 (mouse macrophages) cells [1,4,7,8,9,10,11,12,13,14,15].

A collection of *Stypopodium flabelliforme* from Papua New Guinea showed a different pattern of metabolites with respect to a Chilean one [1,4,5]. This variation in metabolic profile could be linked to the different stage of their life cycle, collection places or/and environmental conditions. On the other hands, both collections produced these three known marker compounds: 2-geranylgeranyl-6-methyl-1,4-benzohydroquinone, stypodiol and stypotriol [1,4,5].

This work describes the isolation and structural elucidation by NMR of a key metabolite in the biogenesis of the meroterpenoids known as taondiol’s family, and the mode of gastroprotective action of the pure compounds epitaondiol and sargaol.

## 2. Results and Discussion

**Figure 1:**
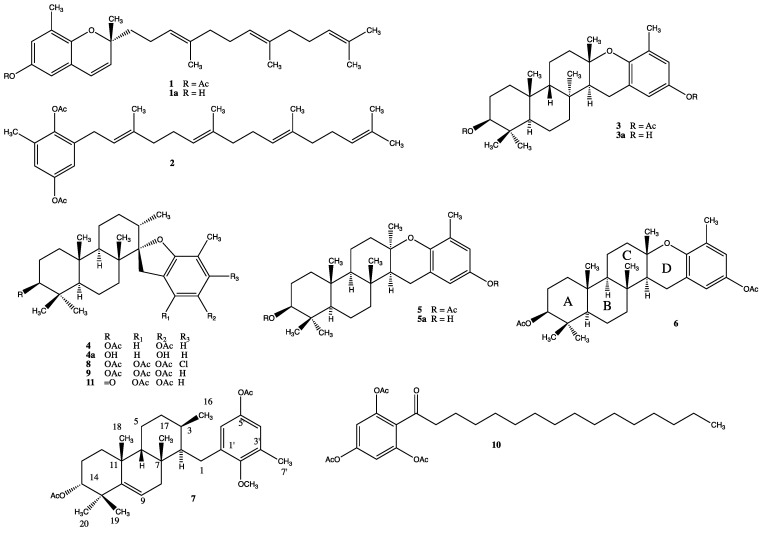
Chemical structures of compounds **1**–**11**.

**Figure 2:**
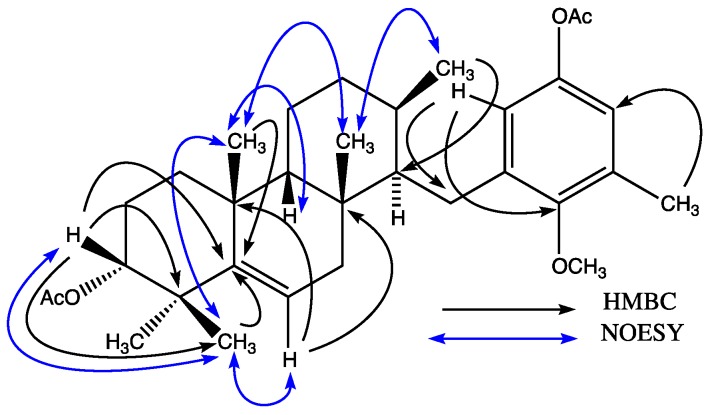
The main correlations in the HMBC (Heteronuclear Multiple Bond Correlation) and NOESY (Nuclear Overhauser Effect Spectroscopy) spectra of **7**.

Compound **7** was isolated as an acetylated derivative from *S. flabelliforme* to avoid the over oxidation of some meroditerpenoids such as stypotriol and tetraprenylhydroquinones (Figure 1). The ^13^C-NMR and mass spectral data indicated that **7** had the molecular formula C_32_H_46_O_5_ indicating ten degree of unsaturation. The ^1^H-NMR spectrum showed signals for two *meta*-coupled aromatic protons at δ 6.64 (d, *J* = 2.8 Hz) and 6.55 (d, *J* = 2.8 Hz), a doublet at δ 5.52 (brd, *J* = 5.5 Hz) assigned to the olefinic proton, a methine proton at δ 4.72 brs assigned to a secondary alcohol, a methoxy group at δ 3.76 s, an aromatic methyl group at δ 2.11 s, five methyl groups at δ 0.75 s, δ 0.80, δ 0.96 d (*J* = 5.7 Hz), δ 1.07 s, δ 1.09 s, and two methyl groups at δ 2.01 s and 2.34 s assigned to the acetyl groups. The ^13^C-NMR spectrum including DEPT 135 (Distortionless Enhancement by Polarization Transfer) showed the presence of eight quaternary carbons (five olefinic), seven methine (two aromatic and an olefinic), six methylenes, six methyl groups, a methoxy, and two acetate groups. Comparison of the spectroscopic data of **7** with those of epitaondiol diacetate and isoepitaondiol diacetate [1,3,5,6] indicated that the ring D is open in compound **7**. This fact was confirmed by HSQC (Heteronuclear Simple Quantum Correlation) and HMBC (Heteronuclear Multiple Bond Correlation) spectra which showed correlations of protons H-6' and H-2 with C-1, and methyl H_3_-16 with C-2, C-3 and C-4 (Figure 2). Further, HMBC cross-peaks between H_3_-18 and C-6, C-10 and C-11, H_3_-19 and C-10, C-14 and C-15, H-9 and C-7, C-8, C-10, C-11 and C-15 allowed the assignment of double bond and secondary alcohol at C-9 and C-14 respectively. Heteronuclear couplings between H-1 and C-1', C-2' and C-6', and between H_3_-7' and C-2, C-3' and C-4' completed the assignment of the aromatic ring. Thus the planar structure of compound **7** was established. Analysis of the NOESY (Nuclear Overhauser Effect Spectroscopy) spectrum clarified the relative configuration (Figure 2). A NOE effect between H-14 and H_3_-19 was observed. H_3_-19 had NOE with H_3_-18, while H_3_-18 showed cross peaks with H-6 and H_3_-17 in the NOESY spectra indicating that these groups are on the same face of the molecule. A similar situation was observed between H_3_-17 with H_3_-16. No NOE cross peak was observed between H_3_-16 and H-2, which implies that both are on the opposite face of the molecule. It is well known that H-14 in epitaondiol, 2β,3α-epitaondiol, isoepitaondiol and taondiol is on the α face of the molecule whose splitting pattern is a doublet doublet (*J* = 11.7; 5.0 Hz) [1,3,4,5,6]. In our case, H-14 is on the β face of the molecule due to its coupling pattern which is a broad singlet; therefore, H-14, H_3_-19, H_3_-18, H-6, H_3_-17 and H_3_-16 are on the β face of the molecule. The above considerations support the proposed unprecedented *syn*-*cis*-*anti* arrangement for the A/B/C ring system for compound **7**. Thus, the structure of compound **7** was elucidated as *O*,*C*(3)-*seco*-9-ene-6β-taondiol.

Other authors have previously reported a possible explanation the biosynthesis of *Stypopodium* meroterpenoids, which may occur thorough the cyclization of 2-geranylgeranyl-6-methylhydroquinone in different folding patterns to give different classes of metabolites related to taondiol’s family [3,4,6,7,16,17]. Our compound isolated is key to a better understanding of the biogenetic pathway, which was suggested for the first time by Gonzalez *et al.* [16].

Previous studies have shown that *Stypopodium zonale* meroditerpenoids included the presence of taondiol, atomaric acid and its three derivatives, stypoldione, stypotriol, epistypodiol, stypodiol, epitaondiol, 2-geranylgeranyl-6-methyl-1,4-benzohydroquinone, 2-geranylgeranyl-6-methyl-1,4-benzoquinone, stypolactone, stypoquinonic acid, 5'a-desmethyl-5'-acetylatomaric acid, and recently zonaquinone acetate, flabellinone, and sargaol [7,18,19,20,21,22]. On the other hands, *Stypopodium flabelliforme* produces epitaondiol, stypotriol, isoepitaondiol, 2-geranylgeranyl-6-methyl-1,4-benzohydroquinone, 2-[2'(*E*)-3',7',11',15'-tetramethylhexadec-2-en-1'-yl]-6-methyl-1,4-benzohydroquinone, stypodiol, epistypodiol, 4'-chlorostypotriol, 14-ketostypodiol, sargaol, 2-(1-oxo-hexadecyl)-1,3,5-trihydroxybenzene, 2β,3α-epitaondiol, flabellinol, flabellinone, stypotriolaldehyde and stypohydroperoxide [1,2,4,5,6]. Contrary to our previous studies, the alga does contain taondiol but not atomaric acid. This is the first report of the presence of taondiol in *Stypopodium flabelliforme*. All compounds were isolated in both species of *Stypopodium* showing that the chemical content may vary depending on the place and time of collection [23].

Among the meroditerpenoids obtained, sargaol **1a** and epitaondiol **3a** (Supplementary Information) have showed gastroprotective activity with ED_50_ values of 35 mg/kg and 40 mg/kg respectively [13]. Oral administration of stypodiol **4a** and isoepitaondiol **5a** at 40 mg/kg inhibited the appearance of gastric mucosal lesions in mice by 69% and 78% respectively. Compounds **9** and **11** were inactive on this model. As shown in Table 1, the compound **4a** and **5a** showed gastroprotective activity in similar way than positive control. The ED_50_ values of **1a** and **3a** were selected for use in the next experiment. To explain the possible mode of gastroprotective action of **1a** and **3a**, we investigated the involvement of prostaglandins (PGs), sulfhydryl compounds (SHs), nitric oxide (NO) and vanilloid receptor (VR) pathway in the protective effects of **1a** and **3a** against HCl/EtOH-induced gastric damage in mice using a pre-treatment with blockers.

**Figure 3:**
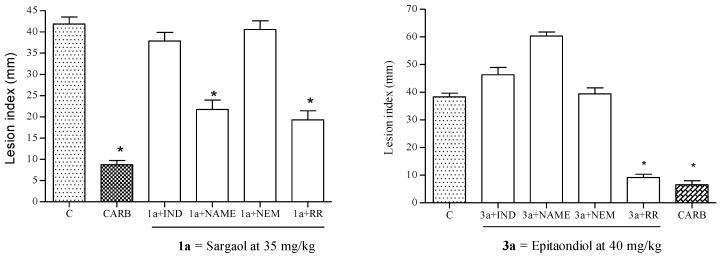
Effect of **1a** on the appearance of gastric lesions induced by HCl/EtOH (*p.o.*) in indomethacin-, *N*-ethylmaleimide (NEM)-, *N*-nitro-l-arginine methyl ester (L-NAME)- and ruthenium red (RR)-pretreated mice. Results are expressed as mean ± s.e.m. *n* = 7. Analysis of variance followed by Dunnett’s test. * *p* < 0.01 compared with the control.

PGs are involved in the protection of the gastric mucosa against necrotizing agents via induction of endogenous PGs [24,25]. In our study, pre-treatment with indomethacin attenuated the gastroprotective effect of **1a** and **3a** (Figure 3). This fact suggested that PGs participate in the protective activity of **1a** and **3a**.

**Table 1:** Gastroprotective effect of compound **1a**, **3a**, **4a**, **5a**, **9**, **11** and lansoprazole at 30 mg/kg on HCl/EtOH-induced gastric lesions in mice and cytotoxicity towards (human epithelial gastric cells (AGS) and human fibroblast.

Compound	*n*	Lesion Index (mm)	% Lesion Reduction	Cytotoxicity IC_50_ (μM)		*p*
				AGS	Fibroblasts	
**1a**	7	20.0 ± 3.6	52 *	18 ± 4	12 ± 3	<0.01
**3a**	7	16.7 ± 3.1	60 *	29 ± 3	19 ± 4	<0.01
**4a**	7	13.0 ± 4.0	69 *	153 ± 9	215 ± 8	<0.01
**5a**	7	9.1 ± 3.6	78 *	42 ± 2	65 ± 3	<0.01
**7**	–	–	–	85 ± 5	102 ± 6	<0.01
**8**	–	–	–	11 ±1	12 ± 1	<0.01
**9**	7	36.4 ± 5.2 **	12	14 ± 3	21 ± 5	<0.01
**11**	7	37.9 ± 7.3 **	9	65 ± 4	102 ± 6	<0.01
Lansoprazole	7	14.1 ± 3.6	66 *	198 ± 5	392 ± 6	<0.01
Control	7	41.5 ± 4.6	–	–	–	–

The results are expressed as mean ± sd * *p* < 0.01; significantly different compared with the control and ** *p* < 0.01 significantly different compared with lansoprazole (ANOVA followed by Dunnett’s test). *n* = number of mice.

Depletion of endogenous SHs has been related with gastric damage induced by ethanol [26]. Endogenous Sulfhydryls such as glutathione is known to protect the integrity and permeability of the cell membrane and may act as antioxidants, scavengers of free radicals, maintenance of immune function, regulation of protein synthesis and degradation, and the maintenance of important surface protein structures [26,27]. In this study, pre-treatment with *N*-ethylmaleimide (NEM, SH-blocker) reduced the gastroprotective effect showed by **1a** and **3a** (Figure 3). This fact indicates that endogenous SHs participate in the protective effect of **1a** and **3a**.

NO participates in gastric defense by the regulation of the gastric mucosal blood flow, angiogenesis and gastric mucus secretion [28,29]. In our study, pretreatment with N^G^-nitro-l-arginine methyl ester (L-NAME, an inhibitor of NO synthase) has not attenuated the gastroprotective effect showed by **1a**. This finding suggests that endogenous NO have null participation in the gastroprotective effect. In the case of the Compound **3a**, the gastroprotective effect was reduced (Figure 3). This fact indicates that endogenous SHs participate in the protective effect of **3a**.

Capsaicin-sensitive sensory neurons via VR on the gastrointestinal tract participate in gastric defense mechanisms (by the regulation of gastric motility, acid secretion, gastric blood flow through the action of calcitonin gene-related peptide (CGRP) and by the stimulation of gastric mucus production and bicarbonate) [30,31]. In this study (Figure 3), pre-treatment with ruthenium red (a vanilloid receptor antagonist), did not reduce the lesion index suggesting that the gastroprotection of **1a** and **3a** have no relationship with capsaicin-sensitive sensory neurons via VR.

Several reviews have discussed the mechanism of action of natural products as gastroprotective agents. For instance, the gastroprotective activity and mechanism of action of the terpenoids aparisthman, *trans*-crotonin, *trans*-dehydrocrotonin carnosol, carnosic acid, cordatin, ferruginol, jatrophone, cyperenoic acid, dehydroabietic acid, imbricatolic acid, horminone, royleanone, solidagenone, suaveolol, azorellane-, and mulinane-type diterpenoids were investigated and discussed [32,33,34,35,36]. In this work stypodiol and isoepitaondiol were investigated for its potential as gastroprotective agents to prevent gastric lesions. Regarding cytotoxic activity the results using the compounds already described correlate well with those previously reported for meroterpenoids, which showed to be cytotoxic against the Caco-2, RBL-2H3, V79, SH-SY5Y and RAW.267 cells [15].

## 3. Experimental Section

### 3.1. Chemicals

TLC (Thin Layer Chromatography) was performed on Kieselgel 60 GF254 using *n*-hexane/EtOAc (8:2 and 1:1 *v*/*v*) as mobile phase. TLC spots were visualized by spraying the chromatograms with H_2_SO_4_-MeOH (5:95, *v*/*v*) and heating at 120 °C for 2–3 min. Column chromatography (CC) was performed over Merck Kieselgel 60, particle size 0.063–0.200 mm. All solvents were dried and purified before use according to standard procedures.

### 3.2. Instrumentation

Measurements of NMR spectra of meroterpenoids used a Bruker Avance AM-400 spectrometer (Bruker, Bremen, Germany) equipped with 5 mm probes. All compounds were individually dissolved in 0.6 mL of CDCl_3_ containing tetramethylsilane (TMS) as internal standard. Chemical shifts (δ) were reported in ppm and coupling constants (*J*) in Hertz. IR spectra were recorded on a Vector 22 FT-IR spectrometer (Bruker, Bremen, Germany). Optical rotations were obtained in CHCl_3_ on a Polax-2L ATAGO, polarimeter (Atago Co., Tokio, Japan). ESIMS (Electrospray ionization mass spectrometry) was recorded on a Waters/Micromass Q-TOF micro high-resolution hybrid quadrupole orthogonal time-of-flight mass spectrometer (Waters Co., Milford, MA, USA) with a constant nebulizer temperature of 100 °C. The experiments were carried out in positive ion mode, and the cone and extractor potentials were set at 10 and 3.0 V, respectively, with a scan range of *m*/*z* 100–600. The samples were infused directly into the ESI source, via a syringe pump, at flow rates of 5 µL min^−1^, through the instrument’s injection valve.

### 3.3. Plant Material

The brown seaweed, *Stypopodium flabelliforme*, was collected by hand using scuba diving near to the coastline of “playa Anakena” in Easter Island, Chile, in May 2013. A voucher specimen (No. SF 15052013) was deposited in the Museo Nacional De Historia Natural, Santiago, Chile while its identity was confirmed by M. Eliana Ramirez from the Museo de Historia Natural de Santiago, Chile.

### 3.4. Extraction and Isolation

Fresh specimens of *S. flabelliforme* (2.0 kg) were frozen for transportation and later extracted with CH_2_Cl_2_ (3 × 2 L). The CH_2_Cl_2_ extract (30.0 g) was then acetylated with Ac_2_O/Py at room temperature and extracted (by using the usual work up) with CH_2_Cl_2_ (3 × 0.5 L) to give 42.0 g of crude extract. The CH_2_Cl_2_ crude extract (41.0 g) was subjected to flash chromatography on silica gel (*n*-hexane/EtOAc; 0% up to 100%) to produce five fractions 1–5. Fraction 1 (*n*-hexane/EtOAc 9:1; 13.0 g), was next applied to a silica gel CC and eluted with EtOAc-*n*-hexane (0.2:9.8, *v*/*v*), and the fractions of interest were further purified by silica gel CC with a *n*-hexane/EtOAc gradient (0% up to 50%) to yield sargaol acetate **1** (280 mg), 2-geranylgeranyl-6-methyl-1,4-benzohydroquinone diacetate **2** (20 mg), epitaondiol diacetate **3** (5 g), stypodiol diacetate **4** (300 mg) and a mixture of meroditerpenoids (500 mg). This mixture was further chromatographed on silica gel CC impregnated with AgNO_3_ (1:9 *w*/*w*) using CH_2_Cl_2_ as mobile phase to give isoepitaondiol diacetate **5** (50 mg), epitaondiol diacetate **3** (110 mg), taondiol diacetate **6** (7mg) and the new compound *O*,*C*(3)-*seco*-9-ene-6β-taondiol diacetate **7** (9 mg).

Fraction 2 (*n*-hexane/EtOAc 7:3; 15.0 g) was subjected to Sephadex LH-20 CC using *n*-hexane/CH_2_Cl_2_/MeOH (3/2/1) to separate chlorophylls and pigments (twice). Fractions of 40 mL were collected and combined for TLC similarity given a fraction (15 g). This fraction was subjected to flash chromatography on silica gel (*n*-hexane/EtOAc, 0% up to 100%) to produce 2 fractions 2A–2C. Fraction 2A (2.0 g) was applied to silica gel CC to yield 4'-chlorostypotriol triacetate **8** (10 mg) and stypotriol triacetate **9** (1.5 g). Fraction 2B (6.0 g) was applied to repeated silica gel CC to give stypotriol triacetate **9** (again, 4.0 g), and 2-(1-oxo-hexadecyl)-1,3,5-trihydroxybenzene triacetate **10** (30 mg). Finally, fraction 2C (1.0 g) was subjected to silica gel CC and subfractions were analyzed by ^1^H NMR with negative results.

Fraction 3 (*n*-hexane/EtOAc 1:1; 10.0 g) was subjected to Sephadex LH-20 using MeOH as mobile phase. Fractions of 40 mL were collected and combined according to TLC similarity to give a subfraction clean (5.0 g). This subfraction was re-chromatographed on silica gel with *n*-hexane and *n*-hexane/EtOAc mixtures of increasing polarity as elution solvents (10% up to 100%) to give stypotriol triacetate (again, 2.0 g) and 14-ketostypodiol diacetate **11** (425 mg).

Fractions 4 (*n*-hexane/EtOAc 3:7; 2.0 g) and Fraction 5 (*n*-hexane/EtOAc 0:1; 1.0 g) were passed through a Sephadex LH-20 column using MeOH. Some of those subfractions were analyzed for ^1^H NMR with negative results for meroterpenoids.

Compounds **1a**, **3a**, **4a** and **5a** were obtained by hydrolysis of **1**, **3**–**5** (see Supplementary Information).

The ^1^H- and ^13^C-NMR data of compound **7** are presented below, whereas the structure of compounds isolated from *S. flabelliforme* is given in the Figure 1.

*O*,*C*(3)-*seco*-9-ene-6β-taondiol diacetate (**7**): white oil; [α]_D_^20^ = −62.0 (*c* 0.5, CHCl_3_); FT-IR ν_max_: 3090, 1750, 1649, 1600, 1430, 1290, 1145 cm^−1^; ESI-MS: calcd. for C_32_H_46_O_5_K [M + K]^+^: 549.8029, found: 549.5176 ^1^H NMR (400.13 MHz, CDCl_3_): 2.48 m; 2.45 m (2H; H-1); 1.24 m (1H; H-2); 1.43 m (1H; H-3); 1.33 m; 1.24 m (2H; H-4); 2.38 m; 2.18 m (2H; H-5); 1.64 m (1H; H-6); 2.40 m; 0.82 m (2H; H-8); 5.52 d (5.5) (1H; H-9); 1.50 m; 1.43 m (2H; H-12); 1.83 m; 1.78 m (2H; H-13); 4.72 brs (1H; H-14); 0.96 d (5.7) (3H; H-16); 0.75 s (3H; H-17); 0.80 s (3H; H-18); 1.07 s (3H; H-19); 1.09 s (3H; H-20); 6.64 d (2.8) (1H; H-4'); 6.55 d (2.8) (1H; H-6'); 2.11 s (3H; H-7'); 3.76 s (OCH_3_); 2.01 s (OAc); 2.34 s (OAc). ^13^C NMR (100.61 MHz, CDCl_3_): 38.7 t (C-1); 42.0 d (C-2); 36.5 d (C-3); 27.4 t (C-4); 22.7 t (C-5); 35.6 d (C-6); 44.2 s (C-7); 35.8 t (C-8); 119.0 d (C-9); 142.9 d (C-10); 44.7 s (C-11); 20.6 t (C-12); 26.2 t (C-13); 78.8 d (C-14); 39.5 s (C-15); 17.5 q (C-16); 17.2 q (C-17); 27.8 q (C-18); 25.0 q (C-19); 27.9 q (C-20); 132.0 s (C-1'); 156.1 s (C-2'); 130.9 s (C-3'); 114.1 d (C-4'); 119.6 s (C-5'); 141.5 d (C-6'); 17.2 (C-7'); 55.3 q (OCH_3_); 169.4 s, 20.9 q (OAc); 170.8 s, 21.2 q (OAc).

### 3.5. Gastroprotective Activity

#### 3.5.1. Chemicals and Drugs

The following drugs were used: Absolute ethanol (EtOH), formalin, Tween 80 and ruthenium red (RR) were purchased from Merck (Darmstadt, Germany). Indomethacin (IND), *N*-ethylmaleimide (NEM), *N*-nitro-l-arginine methyl ester (L-NAME), lansoprazole, and the other chemicals were obtained from Sigma Chemical Co. (St. Louis, MI, USA).

#### 3.5.2. Animals

Swiss albino mice (30 ± 3 g) were purchased from the Instituto de Salud Pública de Chile, Santiago, Chile. Mice were fed on certified Champion diet with free access to water under standard conditions of 12-h dark-light cycle and 22 °C room temperature. The protocols were approved in July 2, 2010 (expiration date: October 31, 2015) by the Ethics Committee of the University of Chile (Chairman Marco Méndez) that follows the recommendations of the Canadian Council on Animal Care and with the ethical guidelines for investigations in conscious animals [37].

#### 3.5.3. HCl/EtOH-Induced Lesions in Mice

The gastroprotective activity of the compounds **4a**–**5a**, and **9**, **11** was assessed in the HCl/EtOH-induced lesion model as described previously [38,39]. Mice were randomly distributed into groups of seven animals each and fasted for 12 h with free access to water prior to the experiment. Fifty min after oral administration of the meroditerpenoids (40 mg/kg), lansoprazole (30 mg/kg) or 1% Tween 80 (10 mL/kg), all groups were orally treated with 0.2 mL of a solution containing 0.3 M HCl/60% ethanol (HCl/EtOH) for gastric lesion induction. Animals were sacrificed 1 h after the administration of HCl/EtOH, and the stomachs were excised and inflated by injection of saline (1 mL). The ulcerated stomachs were fixed in 5% formalin for 30 min and opened along the greater curvature. Gastric damage visible to the naked eye was observed in the gastric mucosa as elongated black-red lines, parallel to the long axis of the stomach similar to the HCl/EtOH-induced lesions in rats. The length (mm) of each lesion was measured, and the lesion index was expressed as the sum of the length of all lesions.

#### 3.5.4. HCl/EtOH-Induced Gastric Lesions in Indomethacin-Pretreated Mice

To investigate the involvement of endogenous prostaglandins in the gastroprotective effect of **1a** and **3a**, indomethacin *s.c.* (30 mg/kg, an inhibitor of the prostaglandin synthesis was dissolved in 5% NaHCO_3_) was injected 30 min before administration of **1a**, **3a** or vehicle (IND-treated) [38,39]. Fifty min after oral administration of **1a** (35 mg/kg), **3a** (40 mg/kg) or vehicle, all groups were orally treated with 0.2 mL of a solution containing 0.3 M HCl/60% ethanol (HCl/EtOH) for gastric lesion induction. Animals were sacrificed 1 h after the administration of HCl/EtOH, and the stomachs were excised and inflated by injection of saline (1 mL). The gastric mucosal lesions were induced and the length of gastric lesions was measured as described above.

#### 3.5.5. HCl/EtOH-Induced Gastric Lesions in *N*-Ethylmaleimide (NEM)-Pretreated Mice

To investigate the involvement of sulfhydryl compounds (SHs) in the gastroprotective effect of **1a** and **3a**, NEM *s.c.* (10 mg/kg, an SH blocker was dissolved in saline) was injected 30 min before administration of **1a**, **3a** or vehicle (NEM-treated) [38,39]. Fifty min after oral administration of **1a** (35 mg/kg), **3a** (40 mg/kg) or vehicle, all groups were orally treated with 0.2 mL of a solution containing 0.3 M HCl/60% ethanol (HCl/EtOH) for gastric lesion induction. Animals were sacrificed 1 h after the administration of HCl/EtOH, and the stomachs were excised and inflated by injection of saline (1 mL). The gastric mucosal lesions were induced and the length of gastric lesions was measured as described above.

#### 3.5.6. HCl/EtOH-Induced Gastric Lesions in *N*-Nitro-l-Arginine Methyl Ester (L-NAME)-Pretreated Mice

To investigate the involvement of endogenous nitric oxide (NO) in the gastroprotective effect of **1a** and **3a**, L-NAME *i.p.* (70 mg/kg, an inhibitor of NO synthase was dissolved in saline) was injected 30 min before administration of **1a**, **3a** or vehicle (L-NAME-treated) [38,39]. Fifty min after oral administration of **1a** (35 mg/kg), **3a** (40 mg/kg) or vehicle, all groups were orally treated with 0.2 mL of a solution containing 0.3 M HCl/60% ethanol (HCl/EtOH) for gastric lesion induction. Animals were sacrificed 1 h after the administration of HCl/EtOH, and the stomachs were excised and inflated by injection of saline (1 mL). The gastric mucosal lesions were induced and the length of gastric lesions was measured as described above.

#### 3.5.7. HCl/EtOH-Induced Gastric Lesions in Ruthenium Red (RR)-Pretreated Mice

To investigate the involvement of vanilloid receptor in the gastroprotective effect of **1a** and **3a**, RR *s.c.* (3.5 mg/kg, a vanilloid receptor antagonist was dissolved in saline) was injected 30 min before administration of **1a**, **3a** or vehicle (RR-treated) [38,39]. Fifty min after oral administration of **1a** (35 mg/kg), **3a** (40 mg/kg) or vehicle, all groups were orally treated with 0.2 mL of a solution containing 0.3 M HCl/60% ethanol (HCl/EtOH) for gastric lesion induction. Animals were sacrificed 1 h after the administration of HCl/EtOH, and the stomachs were excised and inflated by injection of saline (1 mL). The gastric mucosal lesions were induced and the length of gastric lesions was measured as described above.

### 3.6. Cytotoxicity Assay

The cytotoxic assay expressed as cell viability was conducted using MTT assay method [40]. Cells at a density of 3 × 10^4^ of MRC fibroblasts or AGS cells were plated in 96-well culture dishes. Compounds were assayed at concentrations ranging from 0 up to 500 μM. Incubated at 37 °C in humidified CO_2_ incubator for 24 h. After incubation, various concentrations in DMSO solvent of the compounds were added. Each compound was tested in quadruplicate and repeated three times. After 48 h incubation, assay was stop by adding MTT reagent (3-(4,5dimethylthiazol-2-yl)-2,5-diphenyltetrazolium bromide) and the incubation continue for next 4 h before the addition of MTT stop solution containing sodium dodecyl sulphate (SDS), the incubation continue for next 24 h. optical density was measured using microplate reader at 550 nm. IC_50_ value obtained from the plotted graph between percentage live cells compared to control.

### 3.7. Statistical Analysis

Results of statistical analysis were expressed as the mean ± s.e.m. In all experiments, statistical differences between treatments and their respective control were determined by one-way analysis of variance (ANOVA) followed by Dunnett’s test. The level of significance was set at *p* < 0.01. All statistical analyses were performed using the software GraphPad Prism 5 for Windows.

## 4. Conclusions

A new unusual compound was isolated from *S. flabelliforme* along with ten known compounds, which were identified using mainly NMR. *O*,*C*(3)-*seco*-9-ene-6β-taondiol possesses an unprecedented *syn*-*cis*-anti arrangement for the A/B/C ring system not previously encountered in nature. We have isolated taondiol for the first time from this species. Regarding the mode of gastroprotective action at an oral dose of 35 mg/kg, the gastroprotective activity of **1a** was reversed significantly when animals were pretreated with IND and NEM, which suggests that prostaglandins and sulfhydryl groups are involved in the mode of gastroprotective action of sargaol. Finally, the gastroprotective activity of **3a** was reversed significantly when the animals were pretreated with IND, NEM and L-NAME, which suggests that prostaglandins, sulfhydryl groups, and NO are involved in the mode of gastroprotective action of epitaondiol.

## Supplementary Files

Supplementary File 1
