# Supplementary material for: Seco-Taondiol, an Unusual Meroterpenoid from the Chilean Seaweed Stypopodium flabelliforme and Its Gastroprotective Effect in Mouse Model
Source: Mar Drugs. 2015 Mar 30;13(4):1726–38. doi: 10.3390/md13041726 (PMC4413184; doi:10.3390/md13041726)
Supplement: Supplementary File 1 [file marinedrugs-13-01726-s001.pdf]

Supplementary Information

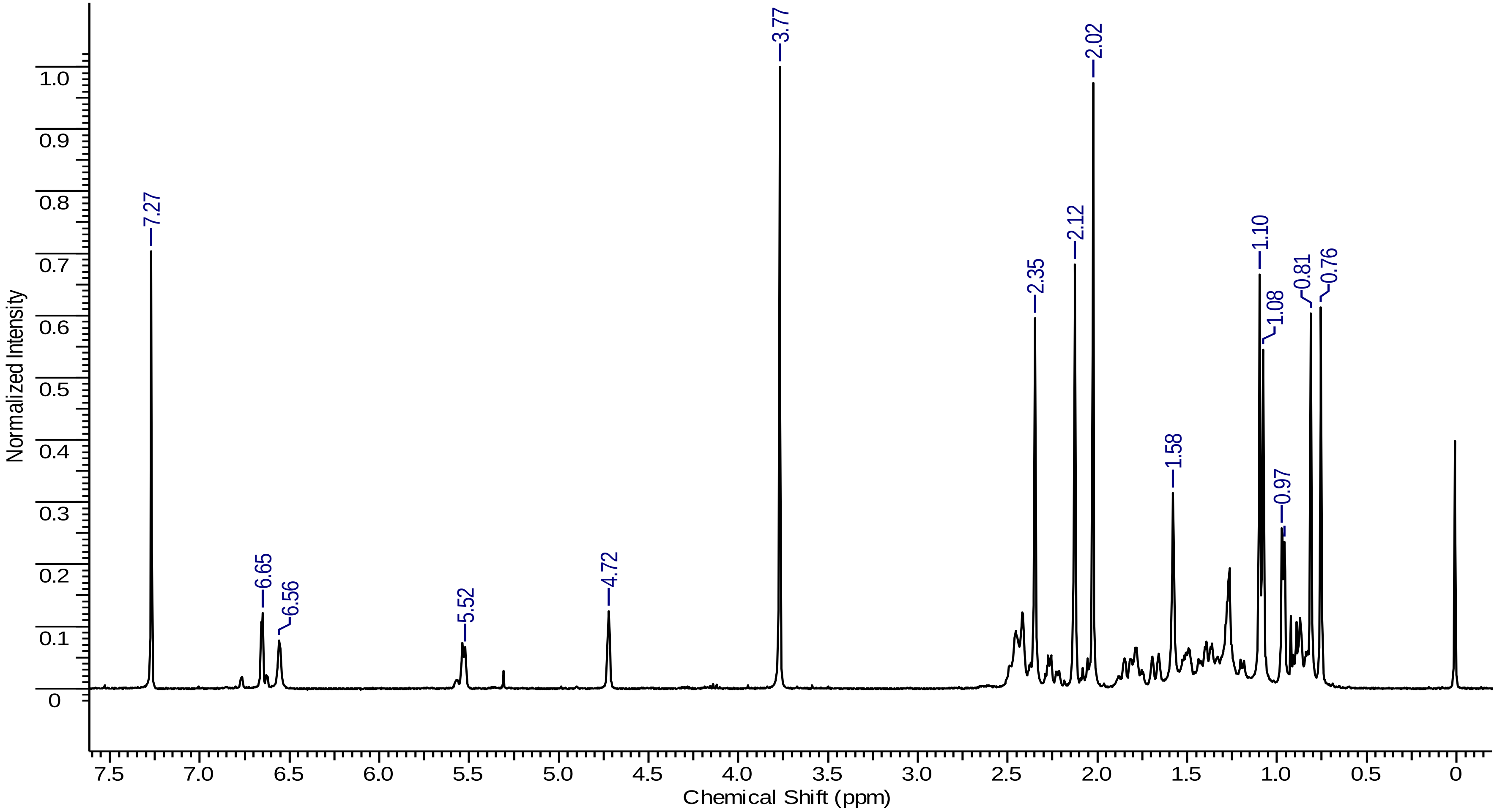

Figure S1. ^{1}H-NMR spectrum of compound **7** ($CDCl_3$).

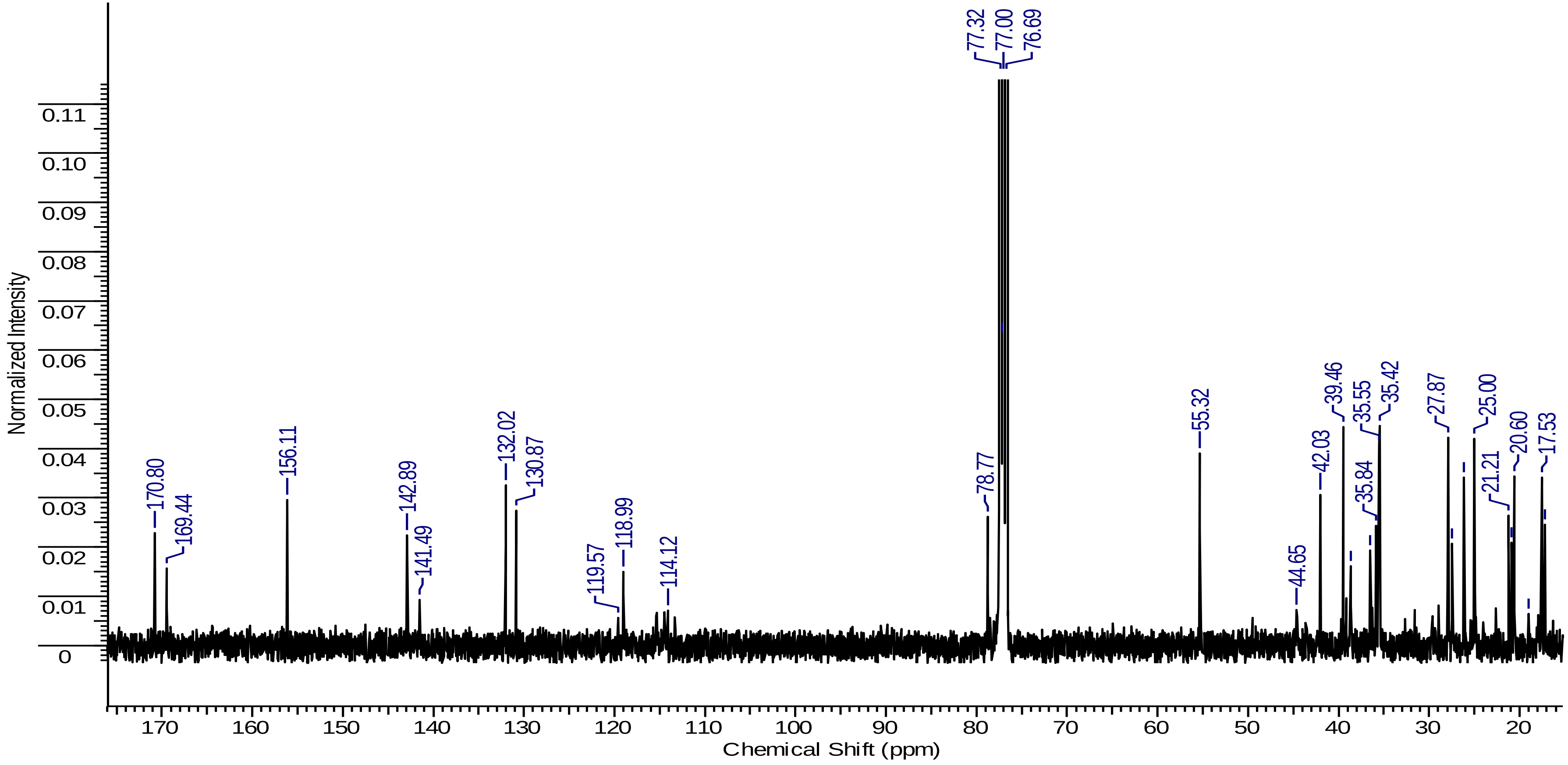

Figure S2. ^{13}C-NMR spectrum of compound **7** ($CDCl_3$).

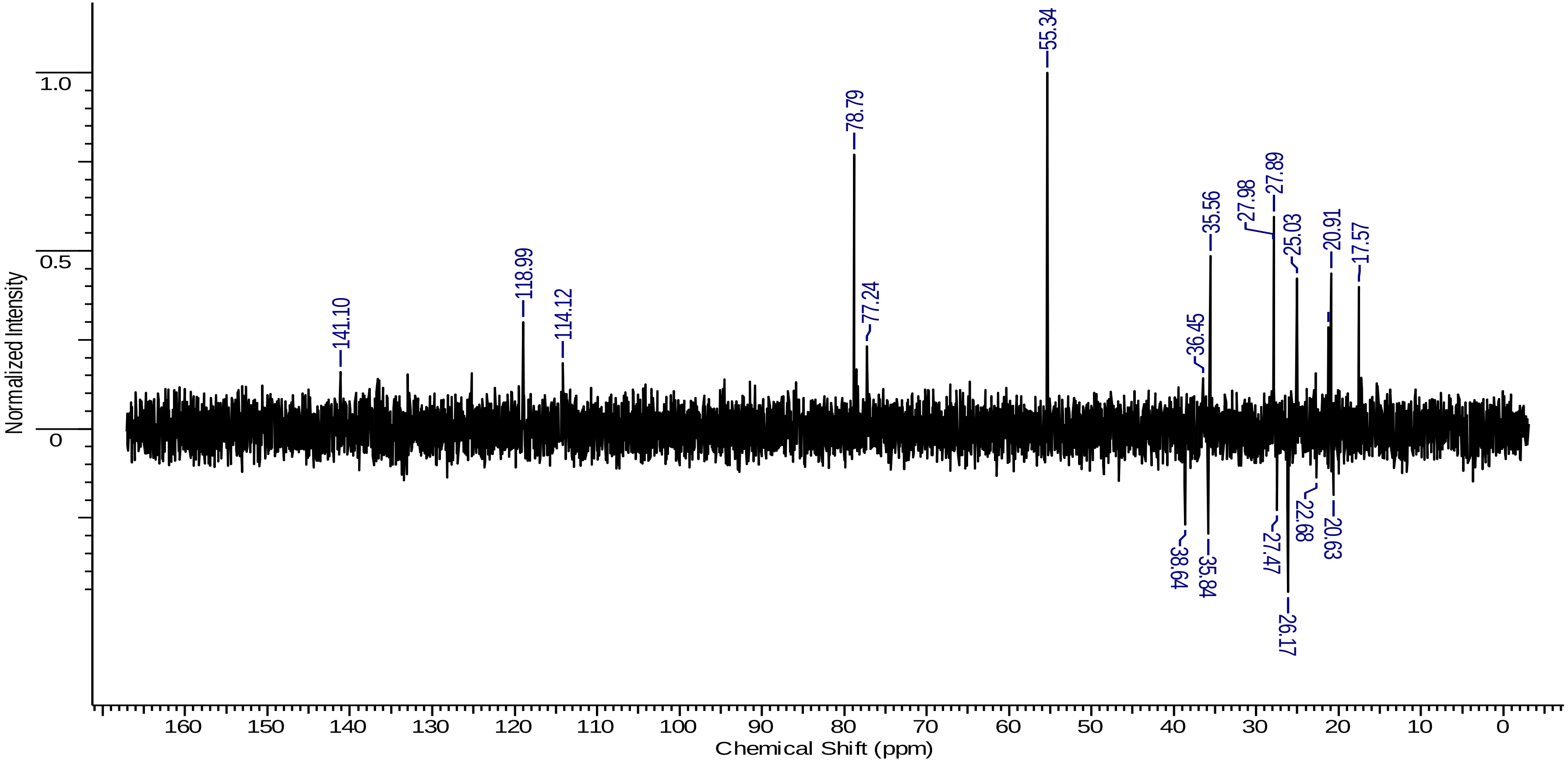

Figure S3. DEPT 135 spectrum of compound **7** ($CDCl_3$).

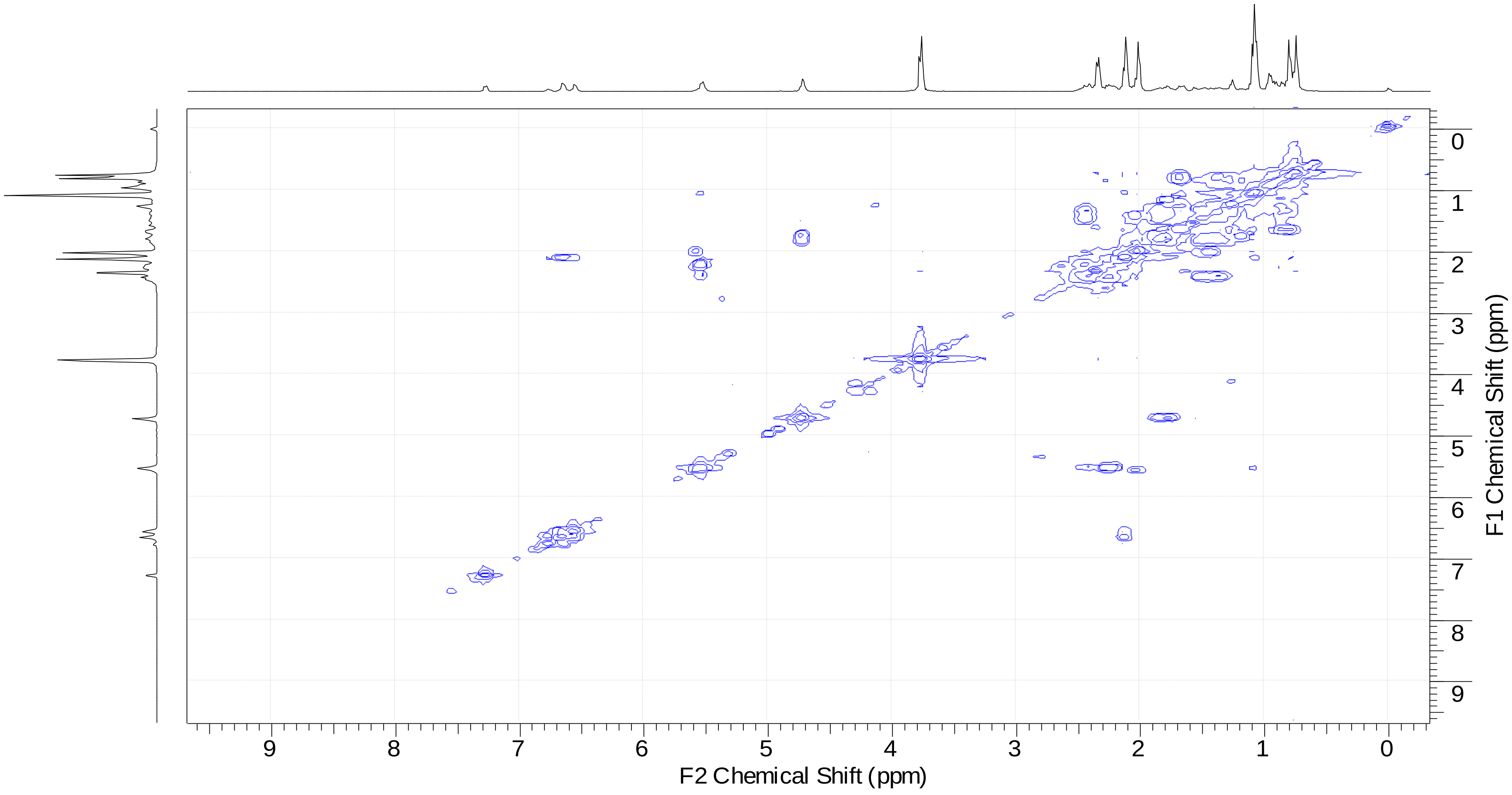

Figure S4. COSY spectrum of compound **7** ($CDCl_3$).

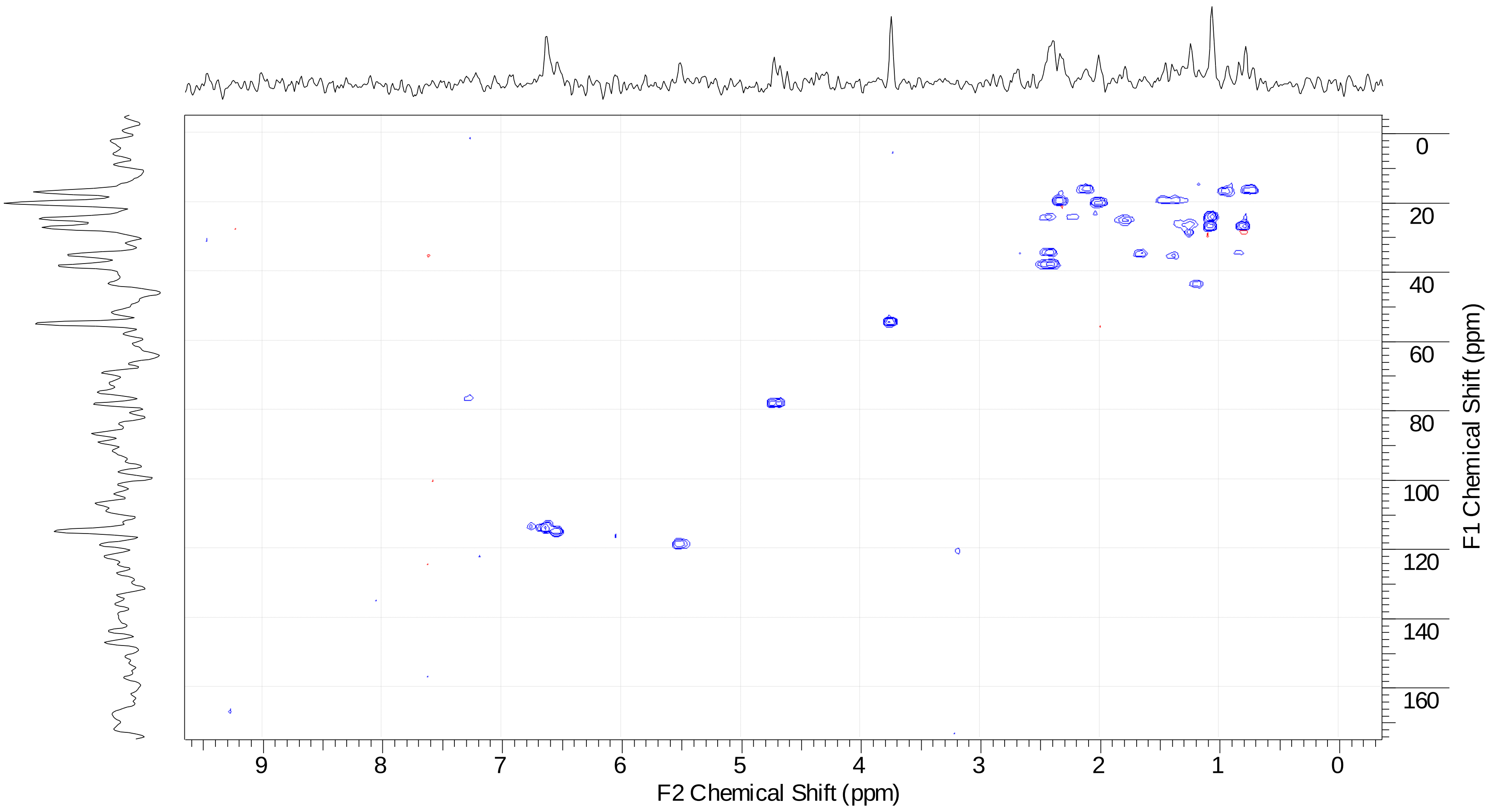

Figure S5. HMQC spectrum of compound **7** ($CDCl_3$).

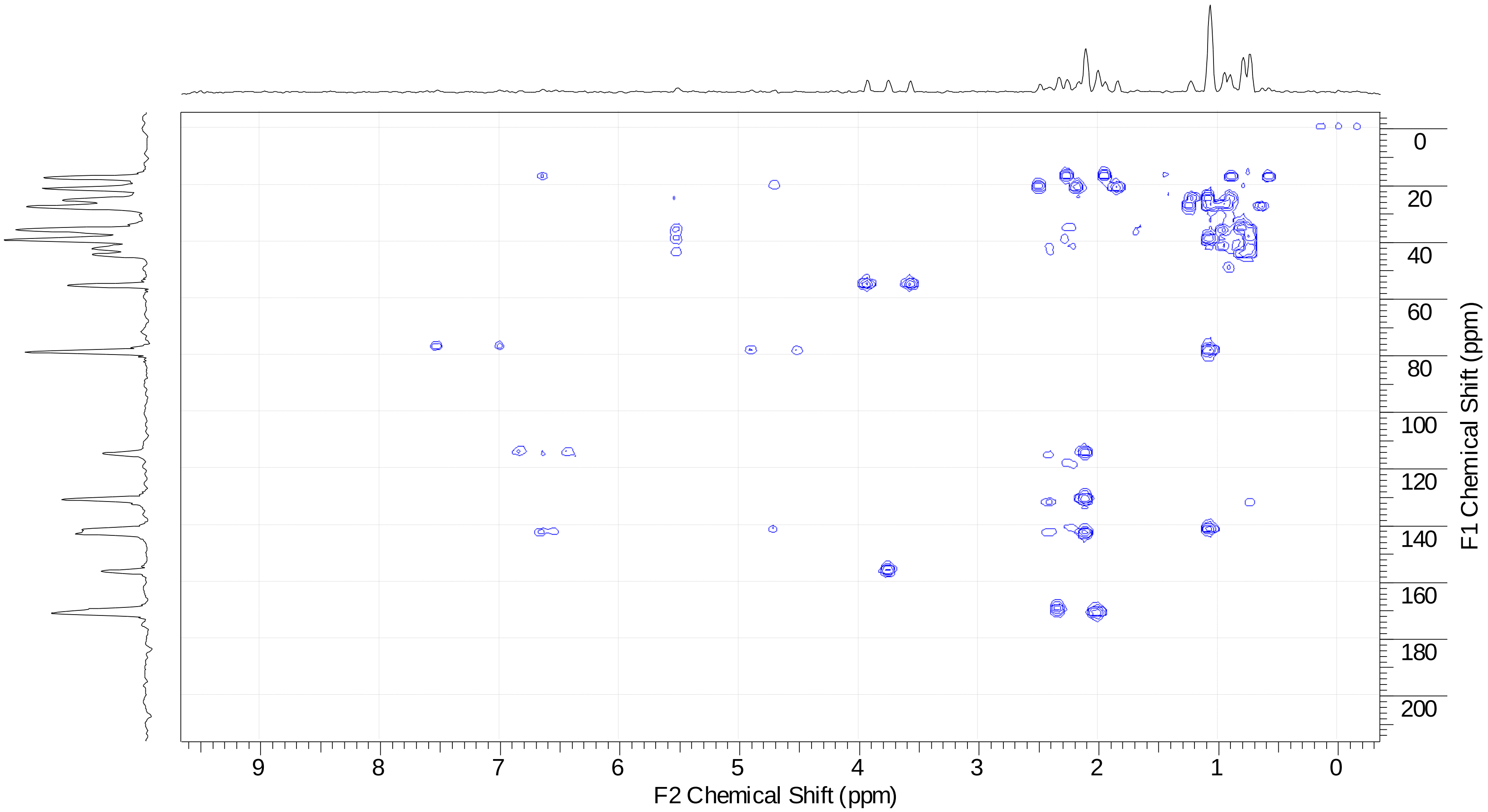

Figure S6. HMBC spectrum of compound **7** ($CDCl_3$).

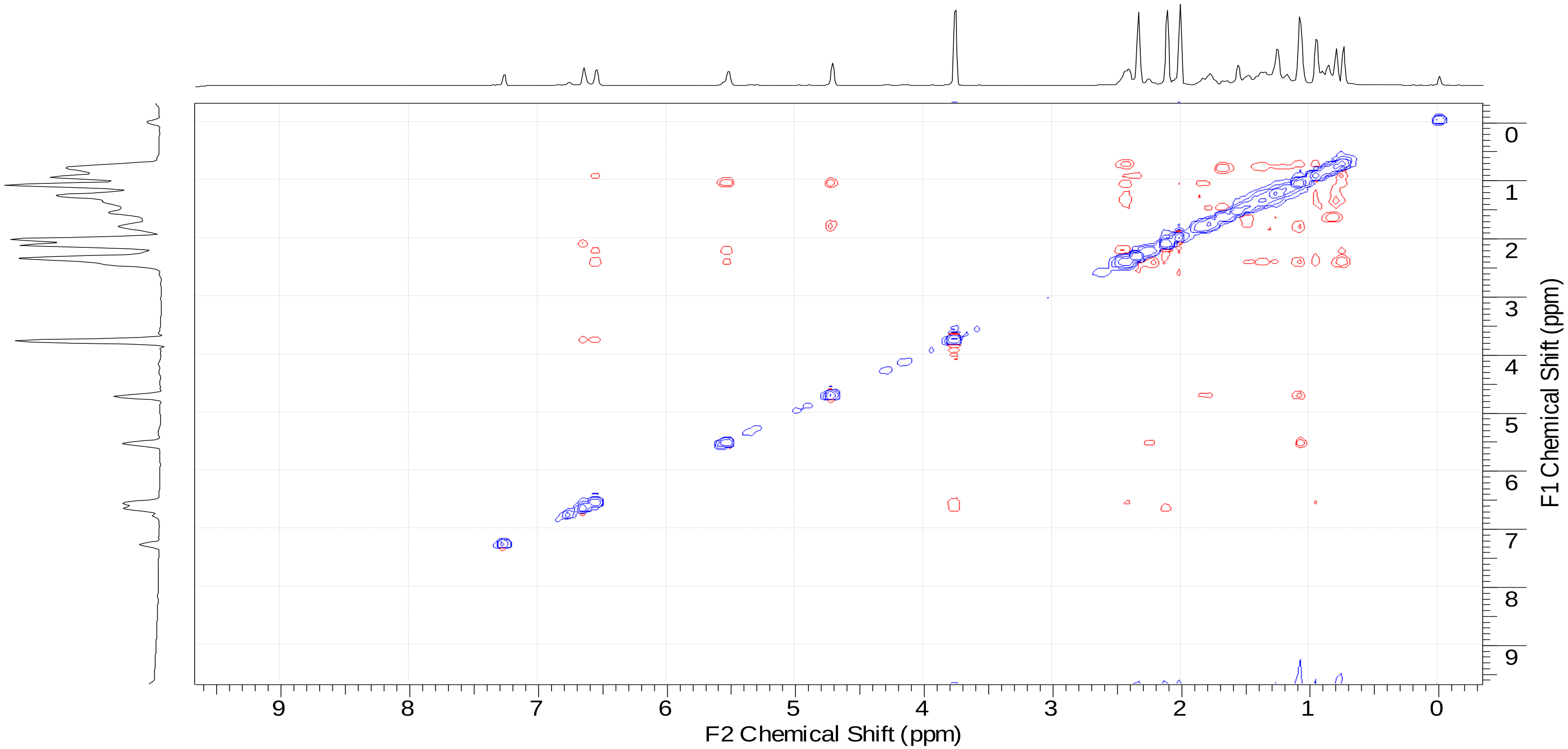

Figure S7. NOESY spectrum of compound **7** ($CDCl_3$).

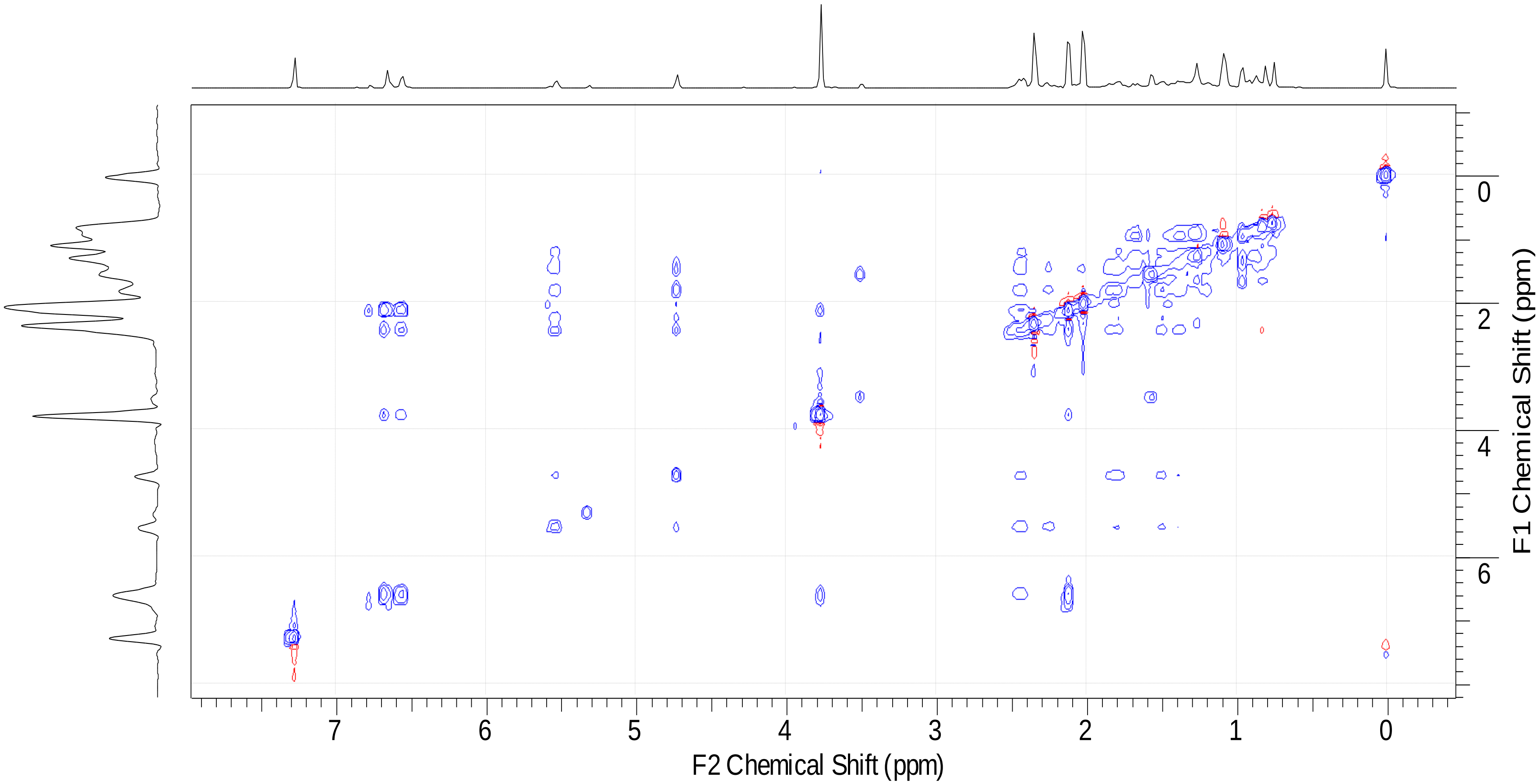

Figure S8. TOCSY spectrum of compound **7** ($CDCl_3$).

General Procedure of Hydrolisis

To a stirred solution of meroterpenoid in MeOH (20 mL), K_2CO_3 (catalytic amount) was added. The reaction mixture was stirred at room temperature under N_2 for 24 h, then rapidly concentrated under N_2 atmosphere. The resulting mixture was then diluted with H_2O (20 mL) and filtered to yield a solid following solvent removed. The latter was re-dissolved in EtOAc for analysis.

© 2015 by the authors; licensee MDPI, Basel, Switzerland. This article is an open access article distributed under the terms and conditions of the Creative Commons Attribution license (http://creativecommons.org/licenses/by/4.0/).
